# Analysis of pH and electrolytes in blood and ruminal fluid, including kidney function tests, in sheep undergoing long-term surgical procedures

**DOI:** 10.1186/s13028-021-00611-0

**Published:** 2021-11-14

**Authors:** Lucie M. Grimm, Esther Humann-Ziehank, Norman Zinne, Patrick Zardo, Martin Ganter

**Affiliations:** 1 Clinic for Swine, Small Ruminants and Forensic Medicine, University of Veterinary Medicine Hannover, Foundation, Bischofsholer Damm 15, 30173 Hannover, Germany; 2 LABVETCON, Laboratory Veterinary Consulting, Föhrenkamp 20, 31303 Burgdorf, Germany; 3 Department of Cardiothoracic, Transplantation and Vascular Surgery (HTTG), Hannover Medical School, Carl-Neuberg-Straße 1, 30625 Hannover, Germany

**Keywords:** Anaesthesia, Beta-hydroxybutyric acid, Bicarbonate, Ovine, Small ruminant

## Abstract

**Background:**

The physiology of sheep as small ruminants is remarkably different from monogastric animals especially regarding the forestomach system. Using sheep for surgical procedures during scientific research thereby presents an exceptional setting for the anaesthetist. Long-term anaesthesia generally demands deprivation of food to reduce the risk of bloat in sheep. This might influence the energy and electrolyte balance. In horses and companion animals, close monitoring of mean arterial blood pressure, capnography and blood gas analysis are common procedures during long-term surgery. However, few data are available on reference ranges for blood gas in sheep and these cover only short periods of anaesthesia. To the authors’ knowledge, there is no study available that includes the monitoring of electrolytes and pH in ruminal fluid and kidney function tests in sheep undergoing long term anaesthesia. Thereby, the aim of the present study was to gather data on blood parameters, and data on ruminal fluid and kidney function during long-term anaesthesia in sheep. Data were obtained from eight sheep undergoing the invasive surgical procedure of left pneumonectomy and auto-transplantation or isolated left lung perfusion. After a 19-h fasting period, the animals were administered xylazine and ketamine and then intubated and maintained in general anaesthesia under artificial ventilation using isoflurane in oxygen. Blood samples were evaluated during 9 h of anaesthesia; ruminal fluid and kidney function tests were evaluated during 7 h of anaesthesia.

**Results:**

Blood parameters such as electrolytes and partial pressure of carbon dioxide revealed few changes, yet blood glucose decreased and beta-hydroxybutyric acid increased significantly. All animals showed an elevated arterial pH and bicarbonate concentration despite artificial ventilation. In ruminal fluid, the pH significantly decreased and no significant changes in electrolytes occurred. Kidney function tests revealed no significant changes in any of the animals. However, fractional excretion of water and phosphate was slightly increased. One animal showed severe complications due to hypokalaemia.

**Conclusion:**

Invasive surgery under long-term anaesthesia in sheep is possible without great imbalances of arterial pH and electrolytes. Nevertheless, potassium concentrations should be monitored carefully, as a deficiency can lead to life-threatening complications. The operated sheep tended not to develop metabolic acidosis and the mean kidney function could be maintained within the physiological range throughout anaesthesia. However, slight elevations in renal fractional water and phosphate excretion could suggest an early tubular reabsorption dysfunction. In ruminal fluid, acidification occurred, though no significant changes were observed in l- and d-lactate levels or in electrolyte concentrations. To our knowledge, the role of the rumen in storing fluids and balancing electrolytes in the blood has not yet been documented during anaesthesia. However, the importance of the rumen for fluid equilibrium in sheep indicates the necessity for routine monitoring and further research.

**Supplementary Information:**

The online version contains supplementary material available at 10.1186/s13028-021-00611-0.

## Background

The physiology of ruminants is remarkably different from monogastric animals, which are widely used in surgical research. This difference is crucial and must be considered by the anaesthetist. Nevertheless, few data on reference ranges for blood gases in sheep are available and these are only covering short periods of anaesthesia [1, 2]. In contrast, to the authors’ knowledge, data on electrolytes and pH in ruminal fluid and kidney function tests are not yet available for sheep undergoing long surgical procedures under general anaesthesia. Despite the differences between sheep and human physiology, the importance of this species to human research becomes clear when reviewing the literature for sheep as models in human research [3–5]. Scientific practice demands that anaesthesia monitoring is an essential element in the success of any surgical study, as it has great influence on the surgical outcome [6]. The American College for Veterinary Anesthesia and Analgesia (ACVAA) recommends close monitoring of the mean arterial blood pressure (MAP), capnography and blood gas analysis during long-term surgery in horses and companion animals [7, 8]. Practical conditions for anaesthesia monitoring in sheep generally differ from those for horses, dogs or humans, as surgery often takes place in the stable or on the field, thereby seldom providing the ideal environment for close monitoring. An exceptional set-up is presented when sheep are used in scientific experimental surgery where interventions are often performed with access to adequate monitoring devices and personnel.

The ovine rumen represents 10–15% of the total body weight [9]. It plays a significant role in storing and absorbing fluids and electrolytes, thereby influencing the electrolyte and acid–base balance of the whole organism [10]. In fact, withholding water for up to 42 h under temperate environmental conditions seems to have no significant cardiovascular effect on sheep [11], which can be related to their great ability to replace extracellular fluid with fluids stored in the rumen [12]. General absorption of electrolytes in the rumen has been described in various in vitro and in vivo studies [10, 13–17]. The rumen acts as a redistribution space for potassium (K) homoeostasis, containing up to 31% of the body’s K content [16]. K adsorption depends on the immediate state of nutrition [14]. In sheep, large amounts of bicarbonate (HCO_3_^−^) [10] sodium (Na) and phosphate (P) [17] are secreted into the rumen via saliva. In Na-depleted animals, K replaces Na in the saliva and may increase up to 100 mmol/L [10]. Regarding the ruminal pH (ru_pH) and K homeostasis during anaesthesia, an influence may occur through the preoperative fasting and the absence of swallowed saliva during anaesthesia. Performing a kidney function analysis in sheep can be carried out using paired blood and urine samples to calculate creatinine clearance (crea_cl) for estimating the glomerular filtration rate and for calculating fractional excretion (FE) rates of several electrolytes and water [18]. The kidney’s influence on fluid and electrolyte balance in the blood is significant [19]. Kidney function is influenced not only by several endogenous factors, especially blood pressure, but also by anaesthesia management and the anaesthetics used [20, 21]. Data on kidney function tests are available on healthy sheep [18], sheep with nephropathies [22] and those with metabolic diseases [23]. Despite the suspected influence of kidney function and ruminal fluid on the general electrolyte status in sheep, so far, no study has monitored these parameters in sheep during surgical procedures under general anaesthesia. Thereby, the aim of this study was to present an anaesthesia suitable for invasive thoracic surgical procedures in order to contribute to existing data on blood parameters and to obtain data on kidney function tests and ruminal fluid during long-term anaesthesia in sheep.

## Methods

### Animals

Sheep entering this study were anaesthetised for performance of left pneumonectomy and autotransplantation or isolated left lung perfusion [24] as a model of alternatives for lung cancer treatment in humans. Animals had to be at least 6 months of age, healthy, determined by means of a thorough clinical examination and female, as urinary catheterisation of the bladder is easier to perform in ewes. All sheep had been bought from a livestock dealer as required by the Federal Ministry of Justice and Consumer Protection [25]. The study was carried out in October and November 2016 and was approved by the Lower Saxony State Office for Consumer Protection and Food Safety (LAVES) under the approval No: 33.012-42502-04-15/1868.

### Housing and feeding

The animals were housed in the stables of the Clinic for Swine, Small Ruminants and Forensic Medicine, University of Veterinary Medicine Hannover, Foundation (Hannover, Germany) where surgery was performed, with a minimum acclimatisation time (30 h) before the beginning of the study. On the day of arrival and directly before surgery, the animals were examined thoroughly by the same veterinarian according to the standard protocol established in the clinic. Visual examined were body posture, behaviour, respiratory rate and type, locomotion. Heart, lungs and rumen were auscultated. Examination of mouth, ears, thorax, abdomen, joints and claws took place, together with an examination of conjunctives, episcleral vessels, mucous membranes, capillary refill time, larynx, and lymph nodes. All sheep were bedded on straw in group stables, hay and water being offered ad libitum and about 200 g of concentrates (Deuka Schaf und Lammfutter, Deutsche Tiernahrung Cremer, Düsseldorf, Germany) being offered per animal daily. The animals were deprived of hay and pellets 19 h prior to the surgical procedure; water intake was not restricted.

### Anaesthesia and preparation

A venous catheter had been inserted into the *vena saphena* (Vasofix® 20G 33 mm, B. Braun Melsungen AG, Melsungen, Germany) of each animal, into which drugs were administered for initial anaesthesia. Premedication took place using xylazine (Xylavet® 20 mg/mL, CP-Pharma Handelsgesellschaft mbH, Burgdorf, Germany; 0.2 mg/kg bodyweight (BW), i.m.), followed after 10 min by ketamine (Ketamin 100 mg/mL, CP-Pharma Handelsgesellschaft mbH; 5 mg/kg BW, i.v.) and if necessary redosed up to three times during preparation with half of the initial doses of ketamine. All sheep were intubated with a cuffed endotracheal tube (Endotracheal PVC Tube, 6–7 mm, Kruuse, Langeskov, Denmark). The urinary bladder was catheterised with a Foley catheter (Foley Ballonkatheter, CH 08, 3–5 mL Ballon, Silcoat®, Jena, Germany) under visual control and an orally placed feeding tube (Levin type 6 mm diameter, 125 cm length, Vygon, Écouen, France) was inserted into the rumen. A permanent thermometer probe was placed into the oesophagus parallel to the feeding tube for body temperature monitoring (Rüsch rectal/pharyngeal temperature probe, Teleflex, Athlone, Ireland) The animals were placed in right lateral recumbency on a foam rubber mat during surgery. A bolus of propofol (Propofol-®Lipuro 10 mg/mL, B. Braun Melsungen AG; 1.8 mg/kg BW, i.v.) was administered once and the anaesthesia was then maintained with isoflurane (Forene 100%®, Ab-Vie Deutschland GmbH und Co KG, Berlin, Germany) at a concentration of 2–2.5% in 50–100% of oxygen using a semi-closed circuit rebreathing system (Model CATO®, Drägerwerk AG & Co. KGaA., Lübeck, Germany). Atracurium (Atracurium Hikma, Hikma Pharma GmbH®, London, UK; 0.9 mg/kg BW, i.v.) was administered for muscle relaxation and the animals were then artificially ventilated at a rate of 16 (± 3) breaths per minute, a positive end-expiratory pressure (PEEP) of 5 (± 1) mbar and a tidal volume of 10 mL/kg BW. A venous catheter was inserted into the *vena jugularis* under sterile conditions (7 Fr. Multi-Lumen-ZVK, BR-24703, Arrow International Inc., Cleveland, OH, USA). For monitoring the mean arterial blood pressure (MAP), a catheter was placed into the left *arteria carotis communis* (Arterial Puncture System, SAC01618, Wayne, PA, USA) and connected to a transducer (DPT6000, Codan Pvb Critical Care GmbH, Forstinning, Germany).

During the operative procedure, heparin (Heparin-Natrium-250000-ratiopharm®, ratiopharm GmbH, Ulm, Germany; 120 IU/kg BW, i.v.) was administered, the animals received individual, dropwise administration of 1 mg norepinephrine (Arterenol® 25 mL 1 mg/mL, Sanifo-Aventis,GmbH, Paris, France) in 250 mL saline (NaCl 0.9%, 200 mL, B. Braun Melsungen AG). In addition, they received fluid therapy with isotonic saline (NaCl 0.9%, 500 mL, B. Braun Melsungen AG) at an infusion rate of 3.8 (± 2.7) mL/kg BW/h. Five ewes received hydroxyethyl starch (HES) (Voluven® 6% Infusionslösung, Fresenius Kabi AG, Bad Homburg, Germany) at 2 (± 1.2) mL/kg BW/h and one animal additionally received lactated Ringer’s solution (Ringer- Laktat nach Hartman, B. Braun Melsungen AG) at a rate of 1.3 mL/kg BW/h. The dose volumes were determined individually for each animal based on the judgement of the anaesthetist.

### Pain management

The sheep received meloxicam (Metacam® 20 mg/mL, Boehringer Ingelheim Vetmedica GmbH, Ingelheim am Rhein, Germany; 0.4 mg/kg BW, i.v.) 20 min prior to the surgical procedure and were provided throughout the experiment with butorphanol (Butomidor® 10 mg/mL, Vetoquinol GmbH, Lure, France; 0.08 mg/kg BW, i.v.) or buprenorphine (Buprenovet® Multidose 0.3 mg/mL, Bayer Vital GmbH, Leverkusen, Germany; 0.008 mg/kg BW, i.v.) and redosed after a period of 3 h. Additionally, an intercostal nerve block was performed by injecting procaine (Isocain ad us. vet, Selectavet GmbH, Munich, Germany; 3 to 5 mL per animal) after opening the ribcage.

### Surgical procedure

Anaesthesia was performed by two alternating veterinarians and one technician. The animals were operated by a team of heart-thorax surgeons from the Department of Cardiothoracic, Transplantation and Vascular Surgery, Hannover Medical School (Hannover, Germany). The procedures performed were either a pneumonectomy of the left lung (PLu) or an isolated lung perfusion (ILuP). For the PLu, the bronchus, arteries and veins of the left lung were dissected, the left lung excorporated and connected to an extracorporeal perfusion system. After a period of extracorporeal flushing, the left lung was autotransplanted and the bronchus, arteries and veins were reattached. Using ILuP, the veins and arteries of the left lung were cannulated to allow a perfusion of the lung with the organ remaining in situ. After perfusing the lung through an extracorporeal perfusion system, the cannulas were removed, and the endothelium of the veins and arteries surgically closed. The mean duration of anaesthesia was 7 h 52 min (± 1 h 56 min) with the left lung excluded from the corporal circulation for 3 h 53 min (± 2 h 13 min). All animals were humanely euthanised (Euthadorm® 500 mg/mL, CP-Pharma Handelsgesellschaft mbH; 150 mg/kg BW, i.v.) after completion of the operative procedures as required by the experimental protocol.

### Sampling

The body weight (BW) of all animals was determined directly before surgery. Heart rate (HR) was measured (CH-2-70-02 and CH-2S-70-02, Datex-Ohmeda Inc., Madison, WI, USA) and documented together with MAP at least every hour. Arterial (Safe pico Aspirator 1.7 mL®, Radiometer Medical, Copenhagen, Denmark) and venous (S-Monovette® 2.6 mL Lithium-Heparin, Sarstedt AG & Co. KG, Nümbrecht, Germany) blood samples were analysed at least every hour to monitor anaesthesia. Onsite blood gas analysis of the arterial sample was performed (Rapidlab 1265® Siemens AG, Munich, Germany). The analyser measures pH (art_pH), partial pressure of carbon dioxide (pCO_2_), partial pressure of oxygen (pO_2_), Na, K, chloride (Cl) and calculates the actual HCO_3_^−^ concentration using measured pCO_2_ and art_pH. The venous blood samples were cooled and immediately transferred to the clinic’s laboratory, centrifuged and the plasma further analysed for total calcium (Ca), l-lactate, d-lactate (Uvicon XL®, BioTeck Instruments GmbH, Bad Friedrichshall, Germany), ß-hydroxybutyric acid (ß-HB), glucose (gluc), magnesium (Mg) and phosphate (P) (Cobas Mira Plus®, Roche Pharma (Schweiz) AG, Basel, Switzerland). At hour one, four and seven after induction of anaesthesia, ruminal fluid and urine were sampled (Eppendorf Tubes® 3810X, Eppendorf Vertrieb Deutschland GmbH, Wesseling, Germany), cooled and transferred to the clinic’s laboratory for analysis. Sampling of ruminal fluid took place via orally placed feeding tube, while urine was purchased though the placed urinary catheter. Urine was analysed for total calcium (u_Ca), phosphate (u_P) (Uvicon XL®), sodium (u_Na) and potassium (u_K) (flame photometry Modell 420, Sherwood Scientific Ltd, Cambridge, UK), chloride (u_Cl) (Rapidlab 1265®), creatinine (u_crea) and gamma-glutamyltransferase activity (u_GGT) (Cobas Mira Plus®). Supernatant of ruminal fluid was analysed for total calcium (ru_Ca), phosphate (ru_P) (Uvicon XL®), sodium (ru_Na) and potassium (ru_K) (flame photometry Modell 420), d- and l-lactate (Uvicon XL®), chloride (ru_Cl) (Chloride Analyzer 925, Corning Inc., Corning, NY, USA) and pH (ru_pH) (MColorpHastTM® pH 5–10 pH-indicator stripes, Merck KGaA). Kidney function tests were performed using paired blood and urine samples, and values for creatinine clearance (crea_cl) and fractional excretion rates (FE) for water, sodium, phosphate, calcium and potassium were calculated [18]. The test kits used were as follows: LT-SYS® Glukose Hexokinase, LT-SYS® Magnesium Xylidblau, LT-SYS® Phosphat Molybdatmethode, LT-SYS® GGT, LT-SYS® Calcium oCPC (Labor + Technik, Eberhard Lehmann GmbH, Berlin, Germany) RNBUT (Randox Laboratories Ltd., Crumlin, UK) and creatinine enzymatic PAP (Dialab GmbH, Wiener Neudorf, Austria). Further chemical components were purchased from Sherwood Scientific Ltd., Roche Diagnostics GmbH, Merck KGaA and Carl Roth GmbH + Co. KG, Karlsruhe, Germany.

### Analysis of data

The data were analysed with SAS Enterprise Guide 7.1 for normality interpreting Q-Q-Plot, Boxplot and the Shapiro–Wilk Test. Data was analysed with a one-way ANOVA, Levene’s test and Dunnett’s test (normally distributed data) or Kruskal–Wallis-test (non-normally distributed data). A P value < 0.05 was considered significant. Data were expressed as median [interquartile range] or mean (± standard deviation of mean (SD)) as appropriate. If more than one blood sample was taken per hour, values were averaged and then used for statistical analysis. As the total duration of anaesthesia differed between the animals, the following analysis included blood samples taken within the first nine hours (63 samples were content of statistical analysis), and ruminal fluid and urine samples taken after one, four and seven hours of anaesthesia (23 samples of each, rumen fluid and urine were content of statistical analysis).

## Results

### General data on anaesthesia

Within this study, data of eight sheep of mixed breed (influence of German blackhead, Merino and Cameroon sheep) were analysed. The animals had a mean weight of 31.1 kg (22.5 to 46.5 kg) BW and were seven to eight months old. During surgery, the mean body temperature of 35.9 [35.3–36.7] °C as well as HR with 86 (± 11) beats per minute and mean MAP of 50 [44–57] mmHg revealed no significant changes. All animals showed salivation during anaesthesia.

### Blood parameters

**Fig. 1:**
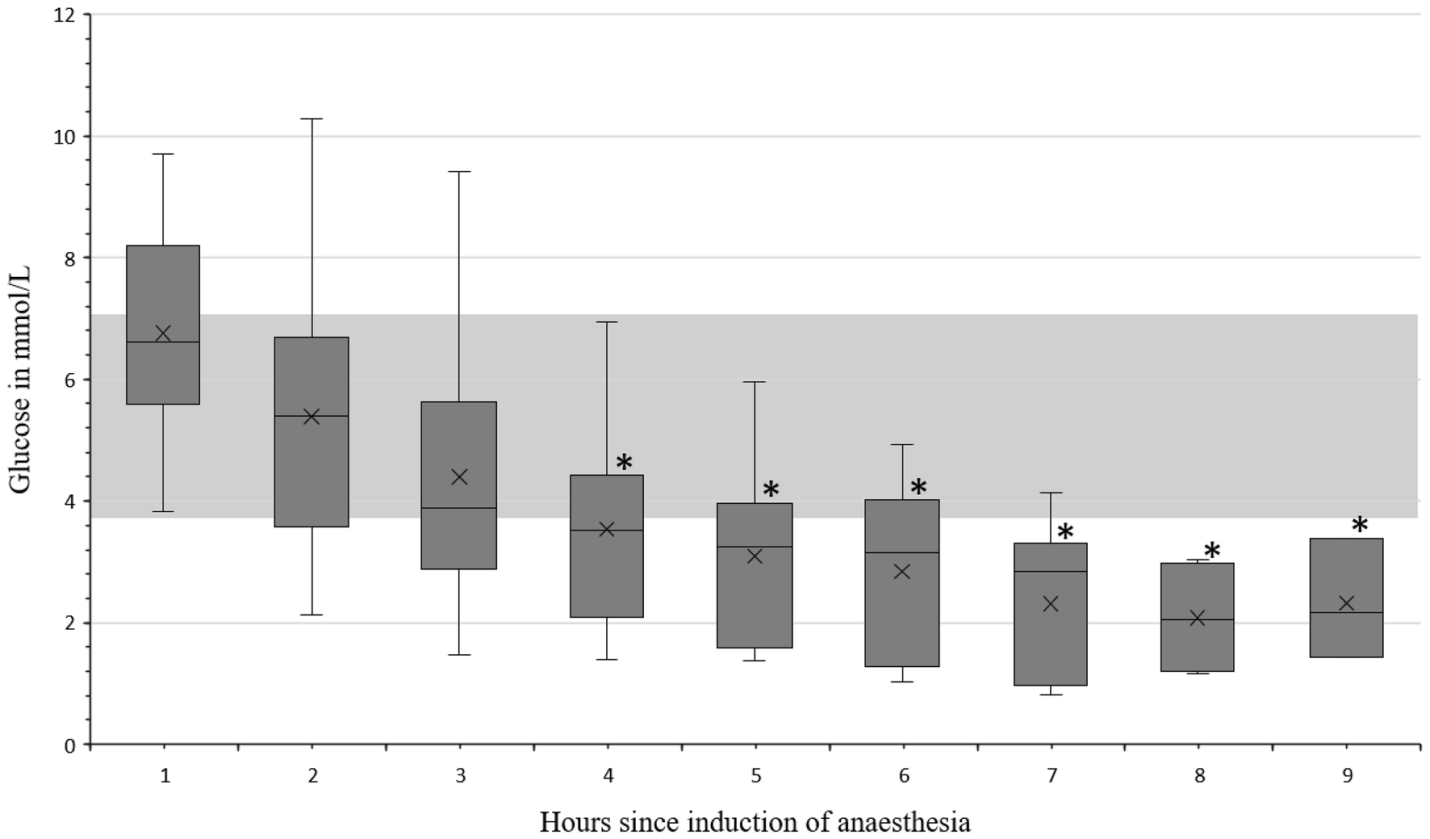
Glucose in the blood of sheep during 9 h of anaesthesia. Data on glucose in the blood of sheep undergoing 9 h of anaesthesia, grey area = reference range [1], * = significant difference to hour 1; hour 4 P = 0.014, hour 5 P = 0.011 h 6 P = 0.009, hour 7 P = 0.008, hour 8 P = 0.023, hour 9 P = 0.041

**Fig. 2:**
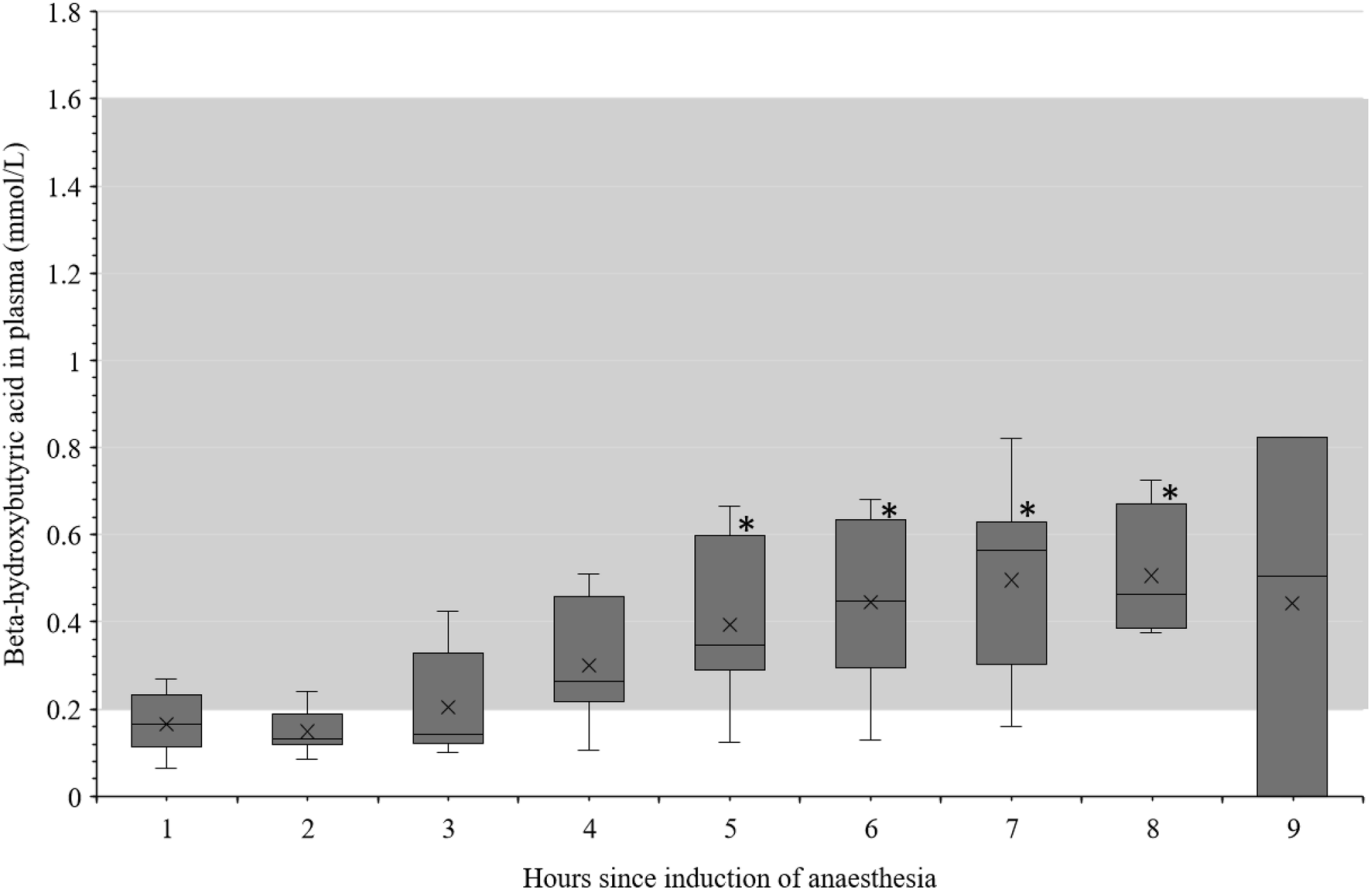
Beta-hydroxybutyric acid (ß-HB) in the blood of sheep during 9 h of anaesthesia. Data on β-HB in the blood of sheep during 9 h of anaesthesia, grey area = reference range [39], * significant difference to hour one; hour 5 P = 0.014, hour 6 P = 0.014, hour 7 P = 0.018, hour 8 P = 0.023

There was a significant decrease in glucose levels in plasma when comparing hour one to hours four to nine (Fig. 1), and in most animals the gluc concentration fell beneath the reference level of 3.1 mmol/L [26] especially at hour eight. ß-HB concentrations never exceeded the reference limits, but a significant increase was observed in the ß-HB concentration in Plasma as visualised in Fig. 2. No significant changes were observed regarding electrolytes in the plasma, but a decrease in the median of Ca of 18% from hour one to seven, and 26% from hour one to nine, resulting in hypocalcaemia, was observed. Data are presented in Additional file 1. The art_pH 7.516 [7.488–7.550] and pCO_2_, 45 [41–47] mmHg did not differ significantly during 9 h of anaesthesia. The pO_2_ was at 182 [133–214] mmHg, inspiratory administered oxygen varied between 50 and 100%. HCO_3_^−^ concentration was 33.8 [32.9–36.2] mmol/L and did not change significantly over time. l-lactate 1.2 [1.0–1.6] mmol/L and d-lactate 0.01 [0.0–0.02] mmol/L levels were not elevated throughout anaesthesia and no significant changes could be observed. One animal showed a life-threatening ventricular fibrillation due to a hypokalaemia (2.72 mmol/L). After administration of a total of 30 mL potassium chloride (Kaliumchlorid 7,45%, B. Braun Melsungen AG; i.v.; 1 mL ≙ 1 mmol K +) the animal showed no further complications during the remaining time of anaesthesia and the potassium levels returned to a physiological range.

### Parameters in the ruminal fluid

**Fig. 3:**
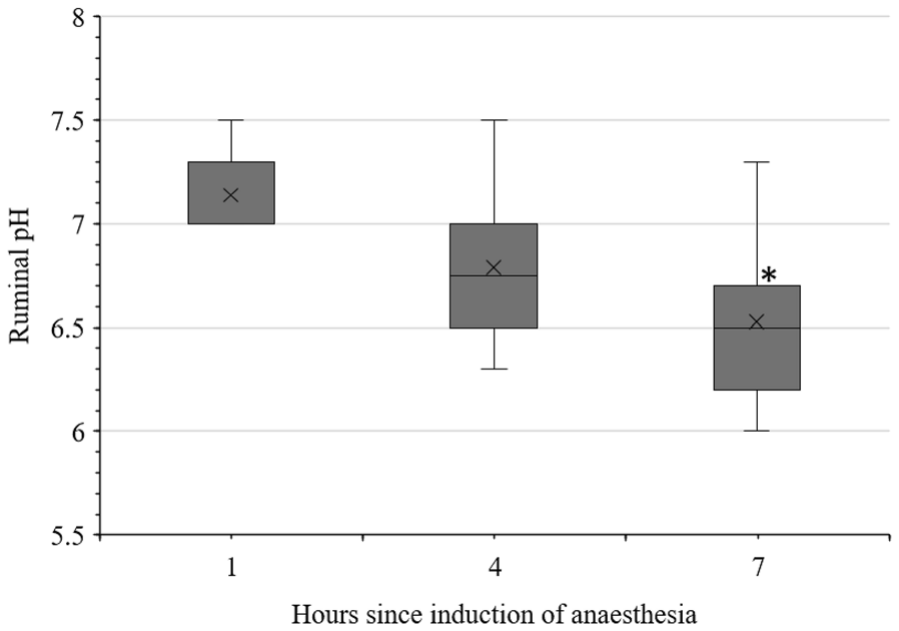
pH in the ruminal fluid of sheep during 7 h of anaesthesia. Data on pH in the ruminal fluid of sheep during 7 h of anaesthesia, * significant difference to hour one, P = 0.023

In ruminal fluid, a significant decrease in pH was observed, as visualised in Fig. 3, while the concentration of l-lactate (0.16 (± 0.05) mmol/L) and d-lactate (0.15 [0.13–0.16] mmol/L) did not change significantly. Electrolytes in ruminal fluid are listed in Table 1. No significant changes were observed, though a rise of 100% in the median of ru_Ca from hour one to hour seven was detected.

**Table 1:** Parameters in ruminal fluid during 7 h of anaesthesia

Variable	Time since induction of anaesthesia (h)		
	1	4	7
pH	7 [7–7.3]	6.75 [6.5–7]	6.5* [6.2–6.7]
Sodium in mmol/L	115.1 [108.2–120.8]	108.6 [96.4–114]	109.9 [94.8–114.4]
Potassium in mmol/L	22.75 [19.9–23.4]	22.9 [20.28–23]	23.4 [20.4–25.8]
Chloride in mmol/L	10 [10–11.8]	14.5 [11.3–18.3]	11 [10–12]
Total calcium in mmol/L	0.98 [0.75–2.66]	0.96 [0.6–1.79]	1.96 [1.2–2.69]
Phosphate in mmol/L	22.11 [20.26–30.01]	21.98 [20.57–31.27]	23.24 [19.17–34.4]
l-Lactate in mmol/L	0.16 ± 0.05	0.15 ± 0.05	0.16 ± 0.05
d-Lactate in mmol/L	0.15 [0.13–0.17]	0.16 [0.13–0.18]	0.13 [0.13–0.23]
No. of individuals	n = 8	n = 8	n = 7

Data expressed as mean ± standard deviation or median [interquartile range]

* Significant difference to hour one, P = 0.023

### Parameters in urine and kidney function tests

The electrolytes in urine did not change significantly; u_GGT was not elevated and showed no significant changes but a rise in the median of u_Ca of 61% was observed from hour one to hour seven; detailed data are presented in Additional file 2. Crea_cl and FE of water, as was the case with FE of Na, P, Ca, and K, did not change significantly. However, a constant decrease in the mean of FE calcium and FE potassium from hour one to seven and an elevation in the mean of FE water and FE phosphate above the reference range could be observed. Detailed data are listed in Table 2.

**Table 2:** Kidney function parameters in sheep during 7 h of anaesthesia, using paired blood and urine samples

Variable	Time since induction of anaesthesia (h)			Reference ranges (18)
	1	4	7	
Crea_cl in mL/kg/min	1.78 ± 0.18	1.70 ± 0.29	1.56 ± 0.38	1.14–2.3
FE water in %	2.95° [1.72–3.16]	2.07 [0.88–4.67]	1.91 [0.89–4.19]	0.2–2.5
FE sodium in %	0.95 [0.33–1.75]	1.07 [0.32–2.87]	1.02 [0.11–2.64]	0.04–1.22
FE phosphate in %	4.71° [1.56–10.51]	3.69° [1.44–10.27]	4.39° [0.48–7.55]	0.01–3.39
FE calcium in %	0.90 [0.12–2.55]	0.69 [0.23–1.91]	0.49 [0.26–5.37]	0.03–8.9
FE potassium in %	76.86 [44.31–201.93]	56.32 [29.79–129.21]	33.61 [25.36–99.47]	12.2–96
No. of individuals	n = 8	n = 8	n = 7	–

Data expressed as mean ± standard deviation or median [interquartile range]

° out of reference range

## Discussion

### Anaesthesia

The MAP was noticeably lower compared to the study by Kampmeier et al. [1]. One clinical factor influencing the MAP is the usage of xylazine as an anaesthetic agent in sheep for which a decreased MAP is described [27]. All operated sheep were consistent in this finding, but the absolute pressure values cannot be guaranteed, as the transducer was not zeroed before recording. A PEEP of 5 (± 1) mbar was applied to reduce anaesthesia-induced atelectasis and to improve gas exchange during anaesthesia [28]. As half of the lung was partially excorporated or cannulated and not available for oxygenation, a reduced body temperature was tolerated, as in humans, this provides the ability to lower tissue oxygen need post operatively [29]. Though in the literature, hypothermia is described as a problem especially in juvenile, non-ruminating, small ruminants during anaesthesia [30], the results emphasise the usage of for example heating mattresses in general in sheep if the setting allows, especially during prolonged anaesthesia. Though in this experiment the absolute loss of saliva was not measured, unpublished observations showed a possible loss of saliva up to 700 mL over eight hours of anaesthesia. The loss of electrolytes and buffering substances has to be taken into account.

### Blood

The detected art_pH was in a similar range as previously described for healthy sheep undergoing anaesthesia [1]. Compared to dogs [31, 32], the art_pH is generally higher in sheep, but as Kampmeier et al. [1] detected a high arterial pH also in awake, healthy sheep, this might represent a physiological state in this species [1]. The invasive lung operation was expected to have an effect, but no significant difference for pCO_2_ could be detected throughout the nine hours of anaesthesia despite the left lung not being circulated for a mean of more than three hours. This might be due to the ventilation strategy, as all animals were constantly under artificial breathing. The occurrence of HCO_3_^−^ was generally higher than in other studies in sheep during anaesthesia [1], though no bicarbonate solution was administered by intravenous infusion and only one animal received an infusion containing metabolisable anions such as lactate. A subtraction alkalosis, which could occur by a reflux of Cl to the rumen, would lead to a decrease in Cl in the blood and an increase in Cl in the ruminal fluid, which was not observed. An elevated HCO_3_^−^ concentration can also be caused by hyperventilation, but in these sheep, the pCO_2_ near the upper reference limit [1] made the possibility of this occurring unlikely. Paradoxically, the loss of saliva, and thereby HCO_3_^−^ [10], did not lead to a decrease in HCO_3_^−^ in the blood. Using xylazine as an anaesthetic agent in sheep, increased blood pH and HCO_3_^−^ concentrations are described [27], but it remains unclear whether this effect lasts up to 9 h after administration. The glucose concentration significantly decreased after four hours of anaesthesia, while ß-HB increased significantly after five hours of anaesthesia but never exceeded the reference limits [26]. In ewes, high ß-HB is described to contribute significantly to the reduction of available glucose in the blood [33]. The drop in blood glucose and increase in ß-HB concentration, signals that glucose consumption led to energy production through lipolysis and together with the missing short fatty acids from the rumen to a rise in ketone bodies in the blood. Correspondingly, (especially in young) sheep, blood glucose levels should be checked regularly, and glucose needs to be adequately substituted during long-term anaesthesia. The electrolytes in the blood showed no significant changes, but the decrease in Ca in the blood, together with a rise in u_Ca and ru_Ca show that fluctuations in the electrolyte balance occur. As the infusion regime differed individually within the animals, the data on electrolytes in the blood cannot be generalised. In dogs, together with imbalances in pCO_2_, a metabolic acidosis due to accumulation of l-lactate does occur throughout long-term anaesthesia causing acidosis [31, 32]. However, in these eight sheep, neither l- nor d-lactate (which might be produced by the ruminal bacteria) were elevated nor significantly changed throughout anaesthesia, so that an influence on the art_pH could not be observed. A loss in K via saliva has to be taken into account, yet the reason for the acute decline in plasma K in one animal remains undetermined. Nevertheless, it should be stated that potassium-rich infusions such as full electrolyte infusions might prevent K deficiency and thereby life-threatening complications. The animals operated afterwards received either lactate Ringer’s solution or additional K applications.

### Kidney

The infusion rate of 3.8 (± 2.7) mL/kg BW/h for isotonic sodium solution was below the infusion rate of 5 to 10 mL/kg BW/h for small ruminants [30] or for dogs recommended by the American Animal Hospital Association [34] during surgery. It was kept generally low to avoid infusion-induced lung oedema, an influence of the infusion rate on the MAP cannot be excluded. The electrolytes in the urine and the kidney function test did not differ significantly over time and the crea_cl suggests that the reduced infusion rate, in combination with the low MAP, did not influence the kidney function significantly within seven hours of anaesthesia. An interpretation of the slight elevation of the mean FE water in the first hour and mean FE phosphate above the reference range is difficult. Physiological elevations of FE water up to 3% and elevations up to 230% in FE phosphate do occur in lambs. Elevations of FE water in adult sheep can be observed which seem to be dependent on food intake [18]. In horses, the elevation of FE water together with a high FE phosphate are associated with early tubular reabsorption dysfunctions [35]. In sheep, this occurrence has not been further investigated. Elevation in u_GGT, which can indicate acute renal tubulus degeneration and necrosis [36], was not observed. As the animals were deprived of food, but had unlimited access to water, an increased water intake seems likely, as in fasted animals undergoing surgery, we commonly see a subjectively more liquid ruminal content. We conclude that the absence of significant changes in HR, MAP and parameters of the kidney function test suggest that a reduced infusion rate still ensures sufficient kidney function in sheep. Yet, it remains unclear whether the elevated FE water and FE phosphate could indicate an early tubular reabsorption dysfunction.

### Ruminal fluid

Ru_pH decreased during anaesthesia with a significant difference from hour one to seven. A rise in d-lactate in the ruminal fluid was expected, which can occur due to bacterial production in an acidified climate in the rumen. Interestingly, neither l- nor d-lactate increased in the ruminal fluid or in the blood. This was probably due to the low amount of metabolisable starch after the 19-h period of starvation prior to anaesthesia. It can probably be assumed that lactate production of ruminal bacteria had no influence on ru_pH. Reflux of acid abomasum content could lead to a slight decrease in pH. Yet, we saw no increased Cl concentration as can be detected in the ruminal fluid of cattle with an abomasal displacement as a consequence of a reflux of abomasal ingesta into the rumen [37]. Assuming the concept of ion interaction occurs in ruminal fluid comparable to ion interaction in the blood [38], the decrease in ru_pH might be influenced by an increase in ru_Ca of 100% from hour one to seven. The possible influx of electrolytes becomes apparent, though no statistical significance could be observed. An additional influencing factor leading to a decrease in the ru_pH represents the absence of buffering substances in saliva such as bicarbonate and phosphate. Our own clinical observations showed a loss of saliva of 400 up to 700 mL during 8 h of anaesthesia (unpublished data). Surprisingly, no significant changes in the phosphate balance were detectable in the ruminal fluid or in the blood. Bicarbonate in the ruminal fluid was not determined. The small number of individuals and the variety in the anaesthesia protocol did not provide a sufficient amount of information on the possible influences. However, it should be noted that ruminal changes in ru_pH and electrolytes did occur. Further investigations into ruminal changes during anaesthesia are needed.

## Conclusion

Long-term inhalation anaesthesia in the operated sheep was possible without great imbalances of art_pH and electrolytes. It is likely that high art_ pH physiologically occurs in sheep, and sheep are not prone to metabolic or respiratory acidosis during long-term anaesthesia under artificial ventilation. Even with a relatively low infusion rate, the mean creatinine clearance could be maintained within the reference limits. However, it must be considered, that adding potassium could prevent life-threatening deficiencies. Loss of important electrolytes and buffering substances via saliva must be considered. In rumen fluid, no significant changes in electrolyte concentrations could be observed but a significant acidification was apparent. Deficits in interpreting the results must be acknowledged due to a lack of information on parameters in rumen fluid of sheep during long-term anaesthesia to compare our results to as well as the relatively small sample size in this study. The effect of the rumen on the fluid and electrolyte equilibrium in the blood during anaesthesia remains largely unexplored and therefore represents a relevant field of study for further research.

## Supplementary Information

**Additional file 1.** Electrolytes in the blood of sheep during 9 h of anaesthesia. Electrolytes in the blood of sheep taken during 9 h of anaesthesia reveal no significant imbalances. Data expressed as mean ± standard deviation or median [interquartile range].

**Additional file 2.** Electrolytes in the urine of sheep during 7 h of anaesthesia. Electrolytes in the urine of sheep, taken during 7 h of anaesthesia reveal no significant imbalances. Data expressed as median [interquartile range].

## Data Availability

The data used and analysed during the current study are available from the corresponding author on reasonable request.

## Abbreviations

ß-HB: Beta-hydroxybutyric acid
art_pH: Arterial pH
BW: Body weight
Ca: Total calcium
Cl: Chloride
crea_cl: Creatinine clearance
PLu: Pulmonectomy of the left lung
FE: Fractional excretion
Gluc: Glucose
u_Ca: Total calcium concentration in the urine
u_Cl: Chloride concentration in the urine
u_crea: Creatinine concentration in the urine
u_GGT: Gamma-glutamyltransferase concentration in the urine
u_K: Potassium concentration in the urine
u_Na: Sodium concentration in the urine
u_P: Phosphate concentration in the urine
HCO_3_^−^: Actual bicarbonate
HR: Heart rate/ heart beats per minute
i.m.: *intramuscular*
i.v.: *intravenous*
IU: International units
ILuP: Isolated lung perfusion
K: Potassium
Mg: Magnesium
MAP: Mean arterial blood pressure
Na: Sodium
ru_Ca: Total calcium in the ruminal fluid
ru_Cl: Chloride concentration in the rumen
ru_Na: Sodium concentration in ruminal fluid
ru_P: Phosphate in ruminal fluid
ru_pH: Ruminal pH
pCO_2_: Partial pressure of carbon dioxide
PEEP: Positive end-expiratory pressure
pO_2_: Partial pressure of oxygen
P: Phosphate
SD: Standard deviation
